# Comparative Genome Analysis Reveals the Genomic Basis of Semi-Aquatic Adaptation in American Mink (*Neovison vison*)

**DOI:** 10.3390/ani12182385

**Published:** 2022-09-13

**Authors:** Lidong Wang, Shengyang Zhou, Tianshu Lyu, Lupeng Shi, Yuehuan Dong, Shangbin He, Honghai Zhang

**Affiliations:** 1 College of Life Sciences, Qufu Normal University, Qufu 273165, China; 2 College of Wildlife and Protected Area, Northeast Forestry University, Harbin 150040, China

**Keywords:** semi-aquatic, American mink, genome, mammal, adaptive evolution

## Abstract

**Simple Summary:**

In nature, every animal lives in its suitable environment to support life and reproduction. Due to survival pressure, the ancient ancestors of land animals changed from living in water to living on land through a long time of evolution. But as more and more animals live on land, the pressure to survive increases, and some animals continue to evolve and re-enter the water. Species evolved from water to land, fins became limbs, and re-entering organisms evolved webbing, all in an effort to better adapt to their environment. The American mink’s life is extremely dependent on the water environment, but its external changes are less pronounced than those of other water-dependent animals. Since the limited external changes are not sufficient to indicate that the American mink is a semi-aquatic mammal, we can explore the evidence of aquatic adaptation at the molecular level. Through comparative genomic analysis, we obtained that American mink has certain adaptive evolution to aquatic environment in olfactory, coagulation, immunity and other aspects. The results of this study have important reference significance for exploring biological evolution and species conservation.

**Abstract:**

Although the American mink is extremely dependent on water and has evolved a range of aquatic characteristics, its structural adaptation to water is still less obvious than that of other typical semi-aquatic mammals, such as otters. Therefore, many scholars consider it not to be a semi-aquatic mammal. In order to make the point that minks are semi-aquatic mammals more convincing, we provide evidence at the micro (genome)-level. In particular, we used the genomes of the American mink and 13 mammalian species to reconstruct their evolutionary history, identified genes that affect aquatic adaptation, and examined the evolution of aquatic adaptation. By analyzing unique gene families, the expansion and contraction of gene families, and positive selection genes, we found that the American mink genome has evolved specifically for aquatic adaptation. In particular, we found that the main adaptive characteristics of the American mink include the external structural characteristics of bone and hair development, as well as the internal physiological characteristics of immunity, olfaction, coagulation, lipid metabolism, energy metabolism, and nitrogen metabolism. We also observed that the genomic characteristics of the American mink are similar to those of other aquatic and semi-aquatic mammals. This not only provides solid genomic evidence for the idea that minks are semi-aquatic mammals, but also leads to a clearer understanding of semi-aquatic species. At the same time, this study also provides a reference for the protection and utilization of the American mink.

## 1. Introduction

Semi-aquatic mammals are species that, through some degree of evolutionary adaptation, rely (at least in part) on aquatic habitats for their daily activities, exceeding the basic dietary requirements for water consumption [1]. The Mustelidae family includes several semi-aquatic species from two sub-families: the Lutrinae (otters) and the Mustelidae (minks). Existing minks include the European mink and the American mink. Despite their similar names, the European mink (*Mustela lutreola*) and the American mink (*Neovison vison*) are less closely related than previously thought [2]. The phylogenetic distance between the two species represents their independent evolution of aquatic adaptation. Previous studies have placed minks in the genus *Mustela*, but cytogenetic, morphological, and biochemical data have placed them in the genus *Neovison* [3,4]. The closest relative of the American mink is the sea mink (*Neovison macrodon*), which unfortunately became extinct in the 19th century due to widespread hunting.

Most semi-aquatic mammals have waterproof fur and shortened appendages, as well as modifications to their feet and tail, which help them to swim and dive. Despite spending most of their time in the water, American minks have very limited physical structural adaptations to aquatic environments [5]. Although the toes of American minks are partially webbed, so are those of closely related terrestrial weasels [6]. The cost of mobility for semi-aquatic mammals may be 3 to 4 times higher than that for land mammals and 2.4 to 5.1 times higher than that for marine mammals [7,8]. The American mink has the highest transportation costs of any semi-aquatic mammal, as it uses more muscle power to paddle, using four legs rather than two [9]. The mink’s fur, while thicker than that of its terrestrial cousins, does not provide the same insulation as that of otters and muskrats [10]. Although the American mink is not perfectly adapted to water, it has still evolved some aquatic traits. While mink fur provides the least buoyancy of any semi-aquatic mammal, its undercoat density is higher than that of any of its terrestrial cousins [11]. The mink’s special eye structure includes the presence of a third eyelid, which protects the front of the eye and removes particles from the surface of the eye to aid underwater vision [12]. Minks also use two thermoregulatory strategies to help them adapt to low temperatures: lowering their resting body temperature while in the nest and maintaining a high level of activity, similar to otters [13]. In swimming, the reduction of mink limb length and the use of underwater gliding to reduce swimming efforts may reduce drag to some extent [14,15]. One strategy to avoid depleting oxygen reserves is to dive for shorter periods, preventing the conversion to anaerobic metabolism; for example, minks will make long, shallow dives, in the form of ‘travel dives’ [16].

In general, many structural changes for aquatic adaptation have occurred in American minks, although they are less pronounced than those in other semi-aquatic mammals. The adaptive evolution of a species occurs to better adapt to its specific environment, which is also a hot research field at present. However, we did not find any studies in the literature focused on the molecular mechanism underlying the semi-aquatic adaptation of the American mink. At present, most studies on the adaptive evolution of species use the method of comparative genomics because genome-wide data contains more complete genetic information and results are more reliable than methods that use a single gene or gene family. Genomic evidence of species-adaptive evolution can be found through the study of species-specific genes, positive selection genes, and gene families; for example, adaptive evolution research on ectothermic snakes (*Thermophis baileyi*) [17], Tibetan antelopes (*Pantholops hodgsonii*) [18], sea otters (*Enhydra lutris*) and giant otters (*Pteronura brasiliensis*) [19], American pika (*Ochotona princeps*) [20], and Himalayan marmots (*Marmota himalayana*) [21] used comparative genomics research methods. 

Therefore, to uncover the genomic basis of the limited body structural changes in mink and the as-yet-undiscovered intrinsic changes, we performed a comparative analysis on the genome of the American mink. The microscopic results of this study, combined with previous macroscopic findings on mink, make the argument that the mink is indeed a semi-aquatic mammal more convincing. At the same time, to prevent a tragedy from happening again (bearing in mind the extinction of the sea mink), this study provides a reference for the protection of minks.

## 2. Materials and Methods

### 2.1. Data Collection and Functional Annotation of American Mink Genomes

We evaluated the genome sequences of 14 species (in FASTA format) and genome annotation information (in GenBank format) retrieved from the National Center for Biotechnology Information (NCBI; https://www.ncbi.nlm.nih.gov, accessed on 5 March 2022). This included genome sequence data and structural annotation data, but did not include the functional annotation of genes. Therefore, we used five databases (NR, Non-Redundant Protein Sequence Database; SwissProt, protein knowledgebase; KEGG, Kyoto Encyclopedia of Genes and Genomes; GO, Gene Ontology; and Pfam, Protein Family Database) to functionally annotate the American mink genome, for which we used BLASTX and BLASTN utilities with an e-value threshold of 1 × 10^−5^. We selected 12 species with better genome assembly and annotation quality (BUSCO > 95%) in Carnivora, as well as one primate species (human, as the outgroup) as our study subjects (Table S1).

### 2.2. Gene Family and Phylogenomic Analyses

To identify gene family groups, protein-coding genes from 14 species—American mink (*Neovison vison*), sea otter (*Enhydra lutris*), North American river otter (*Lontra Canadensis*), ermine (*Mustela ermine*), domestic ferret (*Mustela putorius furo*), giant panda (*Ailuropoda melanoleuca*), domestic cat (*Felis catus*), red fox (*Vulpes vulpes*), polar bear (*Ursus maritimus*), cheetah (*Acinonyx jubatus*), pacific walrus (*Odobenus rosmarus*), human (*Homo sapiens*), dog (*Canis lupus familiaris*), and raccoon dog (*Nyctereutes procyonoides*)—were analyzed. The longest transcript in the coding region was retained to reduce redundancy, and the genes encoding polypeptides shorter than 30 amino acids were also abandoned in order to exclude putative fragmented genes. The similarity relationship between all species’ protein sequences was obtained using an all-against-all BLASTP (https://blast.ncbi.nlm.nih.gov/Blast.cgi/, accessed on 15 March 2022) [22] search with a cut-off E-value of 1 × 10^−5^. The alignment with high-scoring segment pairs was conjoined for each gene pair using Solar software [23]. A hierarchical clustering algorithm was applied to group orthologs and paralogs using OrthoMCL software (http://orthomcl.org/orthomcl/, accessed on 15 March 2022) [24], with an inflation parameter of 1.5.

### 2.3. Phylogenetic Reconstruction and Estimates of Divergence Times

Single-copy orthologous genes were aligned using Muscle (http://www.drive5.com/muscle/, accessed on 17 March 2022) [25]. All alignment results were concatenated to a super alignment matrix. Maximum-likelihood (ML) phylogenetic trees based on multiple sequence alignments were generated using RaxML [26]. Divergence times were estimated using single-copy orthologs by the MCMCTree program of PAML (http://abacus.gene.ucl.ac.uk/software/paml.html, accessed on 17 March 2022) [27] with the main parameters as follows: burn-in = 10,000; sample number = 100,000; sample frequency = 2. The time correction points were giant panda (*A. melanoleuca*) and polar bear (*U. maritimus*) (17.8 and 28.9 Mya). The calibration times were selected from the TimeTree database (http://www.timetree.org/, accessed on 17 March 2022) [28]. We also entered the names of the studied species in this database, with the aim of generating a phylogenetic tree from published online data for comparison with our findings.

### 2.4. Gene Family Expansion and Contraction

Gene family evolution is a random model involving birth and death processes in which gene families expand or contract independently along each lineage of the phylogenetic tree. We initially applied the café package (http://sourceforge.net/projects/cafehahnlab/, accessed on 27 March 2022) in the implementation of the maximum likelihood model in order to compare the ancestors and the cluster size differences between each species (increase or decrease) [29]. A *p*-value of 0.05 was used to identify families with size significantly changed for species. We took into account the phylogenetic tree topology and branch length in order to infer the importance of changes in the size of gene families within each branch.

### 2.5. Positively Selected Genes

Single-copy orthologous genes were aligned with Muscle (http://www.drive5.com/muscle/, accessed on 6 April 2022) [25]. To determine whether positively selected genes were restricted to the *N. vison* lineage, the branch-site model (Ma vs. Ma0) in the CODEML program of PAML4 was used, allowing the ω ratio to have more than one change in the target branches. We used *N. vison* as the foreground branch, while other species (*C. lupus. familiaris*, *N. procyonoides*, *V. vulpes*, *M. erminea*, *M. puorius furo*) served as background clades. This analysis does not simply calculate Ka (non-synonymous) and Ks (synonymous), but also calculates Ka/Ks through the likelihood ratio test in order to detect the probability of positive selection. An ω ratio (Ka/Ks) of <1, 1, or >1 indicates purifying selection, neutral evolution, or positive selection, respectively. The likelihood ratio test (LRT) with χ^2^ distribution and the false discovery rate (FDR) verification method were used to determine the statistically significantly models, compared with the null models (Ma0), at a threshold of *p* ≤ 0.05.

### 2.6. GO and KEGG Enrichment Analyses

The cluster graph R package (3.8.1) was used for gene ontology (GO) enrichment analysis of genes or gene families, and the gene length deviation was corrected. KEGG is a database resource that can be used to understand the advanced functions and uses of biological systems, such as cells, organisms, and ecosystems, through large-scale molecular data sets generated from molecular-level information, particularly those obtained by genome sequencing and other high-throughput technologies (http://www.genome.jp/kegg/, accessed on 27 April 2022) [30]. We detected the statistical enrichment of genes or gene families in terms of the KEGG pathway, using the cluster profile R package (3.8.1).

## 3. Results

### 3.1. Data Collation and Gene Family Clustering Analysis

**Figure 1:**
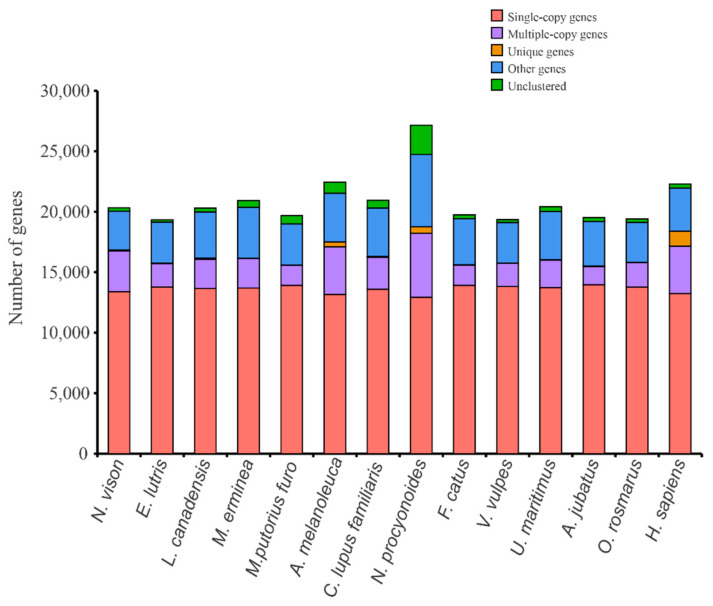
Distribution of orthologous genes in different mammals. Unique genes represent species-specific gene families. Other genes represent gene families outside the above categories.

**Figure 2:**
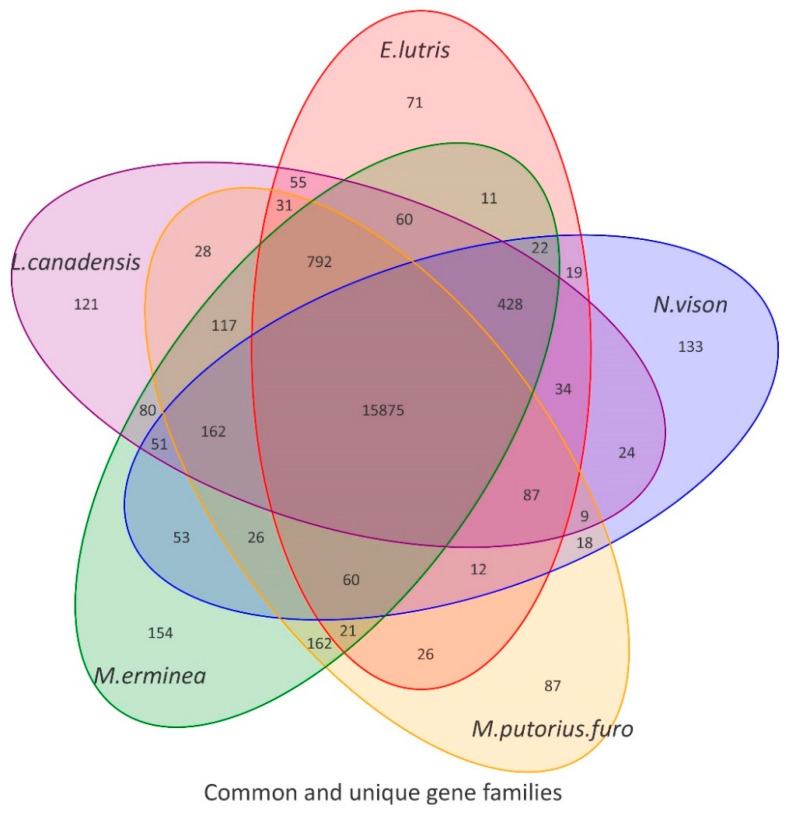
Venn diagrams of the common and unique gene families of five *Mustelidae* species examined in this study.

The number of genes finally used in this project for comparative species genome analysis is presented in Table 1. A total of 20,329 (100%) genes were successfully annotated with putative functions (Table 1). Functional annotation found that 20,325 (99.9%), 20,208 (99.4%), 18,584 (91.4%), 18,591 (91.5%), and 19,300 (94.9%) genes had significant hits with proteins catalogued in the NR, Swissprot, KEGG, Pfam, and GO databases, respectively (Table 1). Cluster analysis of gene families showed that 14 species clustered a total of 20,563 gene families (Figure 1). There were 14,548 gene families, including 9671 single-copy gene families shared by all species. We extracted the corresponding clustering results for the genomes of *N. vison* (American mink), *E. lutris* (sea otter), *L. canadensis* (North American otter), *M. erminea* (stoat), and *M. puorius furo* (domestic ferret) in order to draw Venn diagrams (Figure 2).

As can be seen from the Venn diagram, compared with other species, there were 133 gene families (including 397 genes) that were unique to *N. vison*. Unique gene families are usually associated with the biological characteristics of a species. To understand the genomic basis of the biological characteristics of American mink, we conducted an enrichment analysis of these results. The GO enrichment results of the unique gene families indicated that a total of 38 GO terms (corrected *p* < 0.05) were significantly enriched (Table S2). We note that three terms were related to blood coagulation (GO:0015057, thrombin-activated receptor activity; GO:0070493, and the thrombin-activated receptor signaling pathway; GO:0007596, blood coagulation). Meanwhile, we also noticed that three GO terms (GO:1901566, organonitrogen compound biosynthetic process; GO:0044271, cellular nitrogen compound biosynthetic process; GO:0034641, cellular nitrogen compound metabolic process) were related to nitrogen compound metabolism and synthesis. The KEGG enrichment results of unique gene families indicated that a total of 40 pathways (corrected *p* < 0.05) were significantly enriched (Table S3). Notably, six of these pathways (map04672: Intestinal immune network for IgA production, map04064: NF-kappa B signaling pathway, map04612: Antigen processing and presentation, map04658: Th1 and Th2 cell differentiation, map04659: Th17 cell differentiation, map04660: T cell receptor signaling pathway) were significantly associated with immunity. 

### 3.2. Phylogeny and Divergence

Species differentiation refers to multiple time points, starting from the ancestor nodes, separated by large periods. Thus, to appropriately divide time in a phylogenetic tree, we also need to determine the time correction points, which refer to precise fossil evidence or molecular biological evidence that elucidates the differences in time between identified species and can therefore be used to correct the divergence time calculation in phylogenetic trees. Here, the time correction points were taken from the TimeTree website (http://www.timetree.org/, accessed on 17 March 2022).

**Figure 3:**
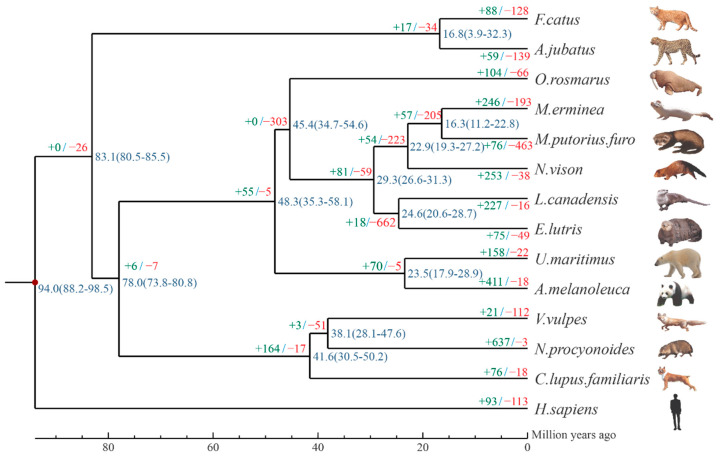
Phylogeny and gene family expansion and contraction analysis of 14 species. Branch numbers indicate the number of gene families that have expanded (green) and contracted (red) after the split from the common ancestor. The time lines indicate the divergence times among the species (blue).

To place our comparative analysis within a well-founded evolutionary framework, we first reconstructed the systematic development history of American mink, as well as that of thirteen other animals (Figure 3), using 9671 single-copy gene families. The phylogenetic tree we constructed includes five species from the Mustelidae family (*N. vison*, *E. lutris*, *L. canadensis*, *M. erminea*, and *M. puorius furo*). Interestingly, American mink shares similar semi-aquatic habits with Lutrinae species, but the phylogenetic analysis suggested that it is more closely related to terrestrial Mustelidae species. In the comparison, it was found that the phylogenetic results of this paper were highly similar to those on the Timetree website and a previous study [31], indicating that our research is highly reliable. We found that the divergence time between Mustelidae and Ursidae was 48.3 million years, slightly longer than that of previous studies (43.8 million years), but still within the 95% confidence interval [19]. For the estimated time of differentiation, the Mustelidae species split into two large branches (Mustelidae and Lutrinae) 29.3 million years ago (Figure 3), sea otters and North American otters split into two branches 27.6 million years ago, and the American mink split from the ancestral species of the Mustelidae 22.9 million years ago, slightly later than the North American otter and sea otter. Domestic ferrets and stoats diverged 16.3 million years ago and are therefore the most recently differentiated species.

### 3.3. Analysis of Gene Family Contraction and Expansion

The results of the expanded and contracted analysis demonstrated that the MRCA (most recent common ancestor) had 20,562 gene families. *N. vison* (American mink) significantly expanded 253 gene families (1308 genes) and contracted 38 gene families (34 genes) compared to their most recent ancestor (Figure 3). 

Further functional enrichment analysis of the expanded gene family revealed 134 significantly enriched gene ontological (GO) terms (corrected *p* < 0.05) and 55 Kyoto Encyclopedia of Genes and Genomes (KEGG) pathways (corrected *p* < 0.05; Tables S4 and S5).

The GO analysis showed that the expanded genes were significantly enriched in the nitrogen cycle (GO:1901566, organonitrogen compound biosynthetic process; GO:0044271, cellular nitrogen compound biosynthetic process; GO:0034641, cellular nitrogen compound metabolic process; GO:1901564, organonitrogen compound metabolic process; GO:0006807, nitrogen compound metabolic process), energy metabolism (GO:0044267, cellular protein metabolic process; GO:0019538, protein metabolic process; GO:0005525, GTP binding; GO:0044260, cellular macromolecule metabolic process; GO:0003924, GTPase activity; GO:0016620, oxidoreductase activity, acting on the aldehyde or oxo group of donors, NAD or NADP as acceptor; GO:0051920, peroxiredoxin activity; GO:0044237, cellular metabolic process; GO:0043170, macromolecule metabolic process; GO:0044238, primary metabolic process; GO:0006183, GTP biosynthetic process; GO:0071704, organic substance metabolic process), blood coagulation (GO:0015057, thrombin-activated receptor activity; GO:0070493, thrombin-activated receptor signaling pathway), and neuropeptide activity (GO:0005184, neuropeptide hormone activity; GO:0004983, neuropeptide Y receptor activity; GO:0008188, neuropeptide receptor activity). Meanwhile, KEGG analysis significantly linked some of the expanded genes to energy conversion (map00010: Glycolysis/Gluconeogenesis) and immunity (map04612: Antigen processing and presentation, map04672: Intestinal immune network for IgA production, map04659: Th17 cell differentiation, map04658: Th1 and Th2 cell differentiation, map04660: T cell receptor signaling pathway, map04621: NOD-like receptor signaling pathway, map04620: Toll-like receptor signaling pathway). 

Further functional enrichment analysis of the contracted gene families stressed four significantly enriched GO terms (corrected *p* < 0.05) and four KEGG pathways (corrected *p* < 0.05; Tables S6 and S7). GO analysis showed that the contracted gene families were significantly enriched in keratin filament (GO:0045095) and olfactory receptor activity (GO:0004984), while KEGG analysis significantly linked some of the contracted genes to olfactory transduction (map04740) and osteoclast differentiation (map04380).

### 3.4. Positive Selection Gene Analysis

With mink as the foreground branch of positive selection analysis and other species (*C. lupus familiaris*, dog; *N. procyonoides*, raccoon dog; *V. vulpes*, red fox; *M. erminea*, stoat; and *M. puorius furo*, domestic ferret) as the background branch, a total of 2315 positive selection candidate genes (q-value < 0.05) were obtained. These positively selected genes were analyzed by Fisher’s test for functional enrichment. Further functional enrichment analysis of the positively selected genes stressed 71 significantly enriched GO terms (*p* < 0.05) and 22 KEGG pathways (*p* < 0.05; Tables S8 and S9).

GO analysis indicated that the positively selected genes were significantly enriched in nitrogen metabolism (GO:0004517, nitric-oxide synthase activity; GO:0006809, nitric oxide biosynthetic process), lipid metabolism (GO:0005543, phospholipid binding; GO:0008289, lipid binding; GO:0045125, bioactive lipid receptor activity; GO:0006665, sphingolipid metabolic process; GO:0038036, sphingosine-1-phosphate receptor activity), energy metabolism (GO:0008422, beta-glucosidase activity; GO:0016709, oxidoreductase activity; GO:0008536, Ran GTPase binding), and physical activity (GO:0005861, troponin complex; GO:0001505, actin filament; GO:0007626, locomotory behavior). KEGG analysis showed that the positively selected genes were significantly enriched in metabolic pathways (map01100), lipid metabolism (map04920: Adipocytokine signaling pathway, map00600: Sphingolipid metabolism, map04071: Sphingolipid signaling pathway), immunity (map04666: Fc gamma R-mediated phagocytosis, map04662: B cell receptor signaling pathway), and phosphonate and phosphinate metabolism (map00440).

## 4. Discussion

We performed a comparative genomic analysis of the genomes of American minks and 13 mammalian species in order to understand the genomic basis for the evolution of semi-aquatic adaptations in the American mink. Genomic evidence of the semi-aquatic adaptation of the American mink was obtained through analysis of unique gene families, gene family expansion and contraction, selection pressure, and phylogenetic differentiation time. We obtained highly reliable phylogenetic trees, and the phylogenetic reconstruction revealed that the closest relatives of the American mink are domestic ferrets and stoats. Although they are most closely related, they diverged very early, which also demonstrates the correctness of its classification in the genus *Neovison* [3]. The results of this study not only considered the external phenotypic structural changes, such as those related to fur, bone, and individual movement, but also included the internal changes related to coagulation, immunity, metabolism, nerves, and olfaction.

Firstly, 20,329 American mink genes were functionally annotated, and the results indicated that all genes were annotated, demonstrating the high quality of the used genome. According to the cluster analysis of the gene families of 14 species, a total of 14,548 gene families were obtained, and 9671 gene families were shared by all species. We found that the unique gene families of the American mink were enriched in GO terms and KEGG pathways related to coagulation, immunity, and nitrogen compound metabolism and synthesis. Blood coagulation in semi-aquatic animals differs from that in terrestrial mammals, in that there is relatively little scab after injury. This reduction in clotting is due to blood contacting water, rather than air [32,33]. The American mink’s unique gene family may enhance its ability to heal after injury, making it more suited to a semi-aquatic lifestyle. As semi-aquatic mammals, American minks may face more challenges from different pathogenic micro-organisms in both terrestrial and aquatic environments [34]. Therefore, the enrichment of unique gene families in immune-related pathways may explain the adaptation of American minks to complex pathogenic micro-organisms in such environments. Although the terrestrial species of Mustelidae have a carnivorous diet, similar to that of the American mink, the diet of the American mink contains a considerable proportion of fish [35]. It is well-known that the protein content of fish is higher than that of other meat, so the unique gene family enriched in GO terms related to nitrogen metabolism may be related to the maintenance of nitrogen balance in the body of the American mink [36]. At the same time, we speculate that this may help American minks to maintain their water–salt balance and osmotic homeostasis, helping to adapt to a semi-aquatic environment [37].

The American mink expanded gene family was mainly enriched in GO terms related to the nitrogen cycle, energy metabolism, coagulation, and nerve activity, as well as KEGG pathways of energy conversion and immunity. In addition to the above discussion, the energy metabolism and conversion, neural activity, and semi-aquatic adaptation of American minks may also be related. A study has shown that increased resistance when swimming at the surface increases energy costs five-fold [38]. Therefore, the expanded gene family of American mink is enriched in the GO terms of energy metabolism and neural activity, which may be related to maintaining fast swimming in the water and improving the hunting success rate. The contracted gene family of American mink was enriched in keratin fiber and olfactory receptor GO terms, as well as osteoclast differentiation and olfactory transduction KEGG pathways. In mammals, according to sequence similarity, OR (olfactory receptor) genes can be divided into Class I and Class II. The former is mainly related to the combination of water molecules, while the latter is mainly related to the combination of air molecules [39]. The contractions of olfactory gene families in American minks are similar to those found in a previous study of sea otters and giant otters. The reason for this may be that American minks spend most of their time hunting in the water and therefore require less of a sense of smell. Although the American mink has a higher density of hair than its terrestrial counterparts, it does not retain heat as well as otter hair does, which may be why the contracted gene family is concentrated in the GO terms of keratin fibers [11]. In addition, the contracted gene family of the American mink was also enriched in the metabolic pathway of osteoclast differentiation, involved in bone formation, which may be related to shaping the bone structure of American mink to adapt to the aquatic environment.

The results of selection pressure analysis indicated that positive selection genes were enriched in the GO terms of nitrogen metabolism, lipid metabolism, energy metabolism, and physical activity, as well as KEGG pathways related to metabolism, lipid metabolism, immunity, and phosphate metabolism. Among these, only physical activity, lipid metabolism, and phosphate metabolism have not been discussed. Phosphate is found mainly in the bones and plays an important role in bone development [40]. Lipid metabolism has a similar function to energy metabolism, which gives the minks sufficient energy to keep swimming and maintain warmth. The GO terms related to physical activity include the troponin complex, actin filament, and locomotory behavior GO terms. American minks need more muscle strength to change from walking on land to swimming in the water, which may be the reason why positive selection genes were found to be enriched in the above GO terms. In conclusion, various physiological behaviors of the American mink may have undergone adaptive evolution in the face of terrestrial and aquatic selection pressures.

## 5. Conclusions

The comparative genomic analysis detailed in this study provides comprehensive insights into the evolution of semi-aquatic fitness in American minks and identifies genetic characteristics relevant to their biology and evolution. The physiological and morphological adaptations of American minks improve our understanding of the functional interactions between behavior, ecology, and genomes. We found that the American mink not only presents semi-aquatic adaptation in terms of its external body structure (e.g., hair, bone, and muscle) but also shows adaptations in its internal physiology (e.g., coagulation, immunity, and metabolism). This study not only provides solid genomic evidence that the American mink is a semi-aquatic mammal but also provides a theoretical basis for the conservation and utilization of wild populations of American mink. 

## Figures and Tables

**Table 1:** The number of genes with functional classification for *Neovison vison*.

Database	Number	Percent (%)
NR	20,325	99.9
SwissProt	20,208	99.4
KEGG	18,584	91.4
Pfam	18,591	91.5
GO	19,300	94.9
Annotation in all databases	18,467	90.8
Annotation in at least one database	20,329	100
Total	20,329	100

## Data Availability

Table S10 provides the NCBI accession numbers of the genomes of the 14 species.

## Supplementary Materials

The following supporting information can be downloaded at: https://www.mdpi.com/article/10.3390/ani12182385/s1, Table S1: Species genome information; Table S2: The GO enrichment analysis of specific gene family in American mink; Table S3: The KEGG enrichment analysis of specific gene family in American mink; Table S4: The GO enrichment analysis of expanded gene families analysis in American mink; Table S5: The KEGG enrichment analysis of expanded gene families analysis in American mink; Table S6: The GO enrichment analysis of contracted gene families analysis in American mink; Table S7: The KEGG enrichment analysis of contracted gene families analysis in American mink; Table S8: The GO enrichment analysis of positive gene analysis in American mink; Table S9: The KEGG enrichment analysis of positive gene analysis in American mink; Table S10: NCBI accession numbers of 14 species.
